# Self‐awareness impairment in Alzheimer’s Disease: Its Impact on Decision‐Making for Aging in Place

**DOI:** 10.1002/alz.088542

**Published:** 2025-01-09

**Authors:** Charline Compagne, Helene Trimaille, Eloi Magnin, Thomas Tannou

**Affiliations:** ^1^ Centre Hospitalier Universitaire de Besancon, Besancon France; ^2^ CMRR, Neurology, CHRU Besançon, Neurosciences intégratives et cliniques EA481, Univ. Bourgogne Franche‐Comté,, Besançon France; ^3^ CRIUGM, CIUSS Centre‐Sud de l'Ile de Montréal, Montreal, QC Canada

## Abstract

Alzheimer’s disease significantly impacts decision‐making abilities, potentially increasing risk‐taking behaviors and complicating the process of aging‐in‐place, particularly in cases of anosognosia (self‐awareness impairment). To understand how this affects patient and caregiver perspectives, we conducted a qualitative study to identify arguments used by older patients with Alzheimer’s disease and their family caregivers about aging‐in‐place. We included 18 dyads, each comprising an older adult living at home with mild dementia and their family caregiver. The older adults' arguments in favor of aging‐in‐place were primarily centered on maintaining a sense of safety and comfort through familiar surroundings and relationships, as well as preserving independence and lifestyle habits, which contribute to stability in their daily lives. Despite recognizing memory loss, many minimized or overlooked the associated risks in their thoughts about aging in place. Caregivers, while supportive of the desire for aging in place, emphasized the need for adaptability in daily functioning and proactive planning for foreseeable challenges. They advocated for external support strategies, including professional assistance, technology use, routine adaptations, and legal protective measures. Our study highlights the profound impact of anosognosia on the loss of adaptability, potentially undermining preventive strategies for aging in place. A deeper understanding of these factors is crucial for professionals in developing care and service plans tailored to individual needs. Such knowledge is vital in facilitating discussions and negotiations around care strategies, ensuring they address the needs and concerns of both patients and caregivers in the context of Alzheimer’s disease.

